# Test anxiety and telomere length: Academic stress in adolescents may not cause rapid telomere erosion

**DOI:** 10.18632/oncotarget.14793

**Published:** 2017-01-22

**Authors:** Yaru Zou, Waiian Leong, Mingling Yao, Xuefei Hu, Sixiao Lu, Xiaowei Zhu, Lianxiang Chen, Jianjing Tong, Jingyi Shi, Eric Gilson, Jing Ye, Yiming Lu

**Affiliations:** ^1^ International Laboratory in Hematology and Cancer, Shanghai Jiao Tong University School of Medicine/Ruijin Hospital/CNRS/INSERM/Nice University, Pôle Sino-Français de Recherche en Sciences du Vivant et Génomique, Ruijin Hospital, Shanghai Jiao Tong University School of Medicine, Shanghai, China; ^2^ Emergency Department, Shanghai Ruijin Hospital North, Shanghai, China; ^3^ Emergency Department, Ruijin Hospital, Shanghai Jiao Tong University School of Medicine, Shanghai, China; ^4^ Dermatology Department, Ruijin Hospital, Shanghai Jiao Tong University School of Medicine, Shanghai, China; ^5^ School of Life Science, Eastern China Normal University, Shanghai, China; ^6^ Xiangming High School, Shanghai, China; ^7^ Exclusive Medical Care Center, Ruijin Hospital, Shanghai Jiao Tong University School of Medicine, Shanghai, China; ^8^ State Key Laboratory of Medical Genomics, Shanghai Institute of Hematology, Ruijin Hospital, Shanghai Jiao Tong University School of Medicine, Shanghai, China; ^9^ Université Côte d’Azur, CNRS, INSERM, IRCAN, Faculty of Medicine, Nice, France; ^10^ Department of Medical Genetics, CHU Nice, France

**Keywords:** adolescent, academic stress, test anxiety, telomere shortening, TL of salivary, Pathology Section

## Abstract

Academic stress (AS) is one of the most important health problems experienced by students, but no biomarker of the potential psychological or physical problems associated with AS has yet been identified. As several cross-sectional studies have shown that psychiatric conditions accelerate aging and shorten telomere length (TL), we explored whether AS affected TL.

Between June 2014 and July 2014, we recruited 200 junior high school students with imminent final examinations for participation in this study. The students were divided into three subgroups (mild, moderate, and severe anxiety) using the Sarason Test Anxiety Scale (TAS). Saliva samples were collected for TL measurement via quantitative polymerase chain reaction (qPCR).

Students from both a specialized and a general school suffered from anxiety (*p* > 0.05). A total 35% had severe anxiety (score: 26.093.87), 33% had moderate anxiety (16.982.64), and 32% had mild anxiety (7.891.92). The TAS values differed significantly (*p* < 0.05) among the three subgroups, but the TLs of saliva cells differed only slightly (*p* > 0.05): 1.140.46 for those with severe anxiety, 1.020.40 for those with moderate anxiety, and 1.120.45 for those with mild anxiety.

Previous reports have found that AS is very common in Asian adolescents. We found no immediate telomere shortening in adolescents with AS. Longitudinal observations are required to determine if TL is affected by AS.

## INTRODUCTION

Excessive academic workloads, fear of failure, emotional exhaustion, and social pressures are routine causes of academic stress (AS) [1]. Whereas modest academic stress encourages persistence and educational success, continuous AS is likely to cause both mental and physical problems. Chronic exposure to emotional and environmental stress is a well-known trigger of physical symptoms, such as increased heart rate, elevated blood pressure, and metabolic disruption. If not treated, these symptoms accelerate the development of cardiovascular disease, diabetes, and other age-related conditions [2–4]. Severe AS is a major health issue for students, but no molecular biomarker of AS or AS-related physical disorders has yet been identified.

Telomeres, which are distinctive nucleoprotein structures capping the ends of linear chromosomes, maintain genomic stability. A telomere is composed of G-rich repetitive DNA protected by a shelterin protein complex that includes telomeric repeat binding factor 1 (TRF1), telomeric repeat binding factor 1 (TRF2), protection of telomeres 1 (protein) (POT1), TRF1-interacting nuclear protein 2 (TIN2), POT1-TIN2-organizing protein (TPP1), and repressor-activator protein 1 (RAP1) [5]. Shelterin not only prevents the unwanted activation of the DNA damage response (DDR) at chromosomal extremities but also facilitates appropriate telomeric DNA replication [6]. Telomeric DNA is also transcribed into a long non-coding RNA, termed TERRA, the function of which remains elusive [7]. Interestingly, an adequate telomere length (TL) not only stabilizes chromosomal ends by allowing the binding of appropriate levels of shelterin but also controls the transcription of genes, even when they are in distant locations from telomeres (i.e., the chromosomal interior) [8]. This “telomere position effect over long distance” (TPE-OLD) appears to involve long-range interactions between the TRF2 shelterin subunit and chromatin [5, 9]. As the brain and central nervous system express large amounts of long non-coding RNAs, including TERRA, and as many genes regulated by TRF2 are neuron-specific, changes in telomere structure (i.e., TL; shelterin and TERRA levels) may play crucial roles in the epigenetic regulation of neuropsychological disorders, highlighting the importance of telomere structure and function in the brain [5].

Of the various telomere parameters, TL is the best-studied biomarker. TL measures cellular replicative capacity; TL is reduced after each round of DNA replication until a critically short TL triggers irreversible cell cycle arrest (senescence) or apoptosis. Such somatic erosion is caused by the absence, or low-level expression, of telomerase in somatic cells. Indeed, a reverse transcriptase ensuring telomere maintenance is active during embryogenesis but becomes inactive after birth [10]. Importantly, the rate of telomere shortening caused by cell division is accelerated by oxidative stress, exposure to genotoxics, and increased stress hormone levels [5, 11, 12]. Recent epidemiological studies have shown that accelerated telomere loss is an early predictor of cardiovascular disease, cancer, type 2 diabetes, and cognitive decline [13]. Thus, as TL is a measure of individual aging, TL may reflect a history of psychological disorder.

Several cross-sectional studies have shown that TL is associated with mental disorders. Patients suffering from major depressive disorder (MDD), generalized anxiety disorder, bipolar disorder, or schizophrenia exhibit more severe telomere erosion in blood leukocytes than do controls [14–16]. Thus, telomeres may link psychiatric conditions to senescence, and TL erosion may increase the incidence of age-related diseases. Notably, hippocampal TL decreased more markedly than did that of other brain regions in patients with MDD [17]. As the hippocampus plays a critical role in neurogenesis and as hippocampal volume is reduced in MDD patients, TL may be involved in the brain aging associated with MDD.

TL is a marker of clinical susceptibility to psychiatric conditions. TL shortening is hereditary in depressed mothers and is particularly evident in families that are fragile in terms of their social environment and level of psychological stress [18, 19]. Thus, TL is a potential biomarker of cumulative exposure to adversity in early life and predicts the long-term risk of psychiatric conditions [16–20]. However, the use of TL as a molecular biomarker of such conditions remains controversial [21]. For instance, gender plays a role in the telomere erosion induced by psychiatric conditions [22, 23].

We examined whether TL was associated with the anxiety levels of healthy students by measuring the TL of salivary cells from students exposed to AS related to the imminence of their final examinations, the most stressful period of the semester.

## RESULTS

### Participant characteristics

Participant characteristics are presented by anxiety level (Table 1a) and school grade (Table 1b). We found no significant differences in any of the baseline demographic characteristics (age, gender, height, weight, or body mass index [BMI]) according to these variables. Furthermore, age, gender, and BMI did not have a significant effect on either the TAS score or salivary cell TL (all *p* values > 0.05).

**Table 1A:** Demographic information of the participants (classified by TAS score)

	Mild anxiety	Moderate anxiety	Severe anxiety	*P* value
**Age(year)**	13.91±0.11	13.87±0.11	13.97±0.12	*p* > 0.05
**Gender (*n*, %)**				
*Male*	30(15%)	36(18%)	34(17%)	*p* > 0.05
*Female*	34(17%)	30(15%)	36(18%)	*p* > 0.05
**BMI(kg/m^2^)**	22.05±1.03	21.58±1.87	22.60±1.02	*p* > 0.05
**Factors controlled for by study design**				
*Current chronic diseas*e^a^	0	0	0	-
*Major depressive disorder*^b^	0	0	0	-
*Smoki*n*g*^c^	0	0	0	-
*Drinking*^d^	0	0	0	-
*Childhood trauma*^e^	0	0	0	-
*Obsteric risk condition during mother's pregnancy*^f^	0	0	0	-
**Test anxiety score**	7.89±1.92	16.98±2.64	26.09±3.87	*P* < 0.05
**Telomere length(T/S ratio)**	1.12±0.45	1.02±0.40	1.14±0.46	*p* > 0.05

**Table 1B:** Demographic information of the participants (classified by school grade)

	Specialized school	General school	*P* value
**Age(year)**	13.41±0.91	13.29±0.48	***p*** > 0.05
**Gender (*n*, %)**			
*Male*	48(48.98%)	49(49.49%)	***p*** > 0.05
*Female*	50(51.02%)	50(50.51%)	***p*** > 0.05
**BMI(kg/m^2^)**	22.60±2.03	21.97±2.83	***p*** > 0.05
**Factors controlled for by study design**			
*Current chronic disease*^a^	0	0	-
*Major depressive disorder*^b^	0	0	-
*Smoking*^c^	0	0	-
*Drinking*^d^	0	0	-
*Childhood trauma*^e^	0	0	-
*Obsteric risk condition during mother's pregnancy*^f^	0	0	-
**Test anxiety score**	21.44±7.77	19.01±7.47	***P*** > 0.05
**Telomere length(T/S ratio)**	1.12±0.48	1.16±0.48	***p*** > 0.05

a *Current chronic disease*: including arthritis, asthma, cancer, COPD, diabetes and viral diseases such as hepatitis C and HIV/AIDS, and the course of the disease lasts for more than three months.

b *Major depressive disorder* diagnosed with DSM-IV MDD, the patient must meet five out of nine symptom criteria: 1) depressed mood most of the day, nearly everyday, as indicated by either subjective report or observation made by others; 2) markedly diminished interest or pleasure in all, or almost all, activities most of the day, nearly everyday (as indicated either by subjective account or observation made by others); 3)significant weight loss when not dieting or weight gain, or decrease or increase in appetite nearly every; 4) insomnia or hypersomnia nearly everyday; 5) psychomotor agitation or retardation nearly every day; 6) fatigue or loss of energy nearly every day; 7) feelings of worthlessness or excessive or inappropriate guilt nearly every day; 8) diminished ability to think or concentrate, or indecisiveness, nearly every day; 9) recurrent thoughts of death (not just fear of dying), recurrent suicidal ideation without a specific plan, or a suicide attempt or a specific plan for committing suicide.

c *Smoking*: subjects were classified as past smoker, or current smoker and the typical number of cigarettes smoked per day and number of years the individual smoked were recorded.

d *Drinking*: subjects were classified as past drinker, or current drinker and the number of alcohol units consumed in a typical week was recorded.

e *Childhood trauma*: the presence of traumatic events in childhood (including death of a close relative, separation from a parent, and sexual or physical abuse).

f *Obsteric risk condition during mother's pregnancy*: exposure to stress or other adverse conditions during intrauterine development.

### Anxiety levels

**Figure 1:**
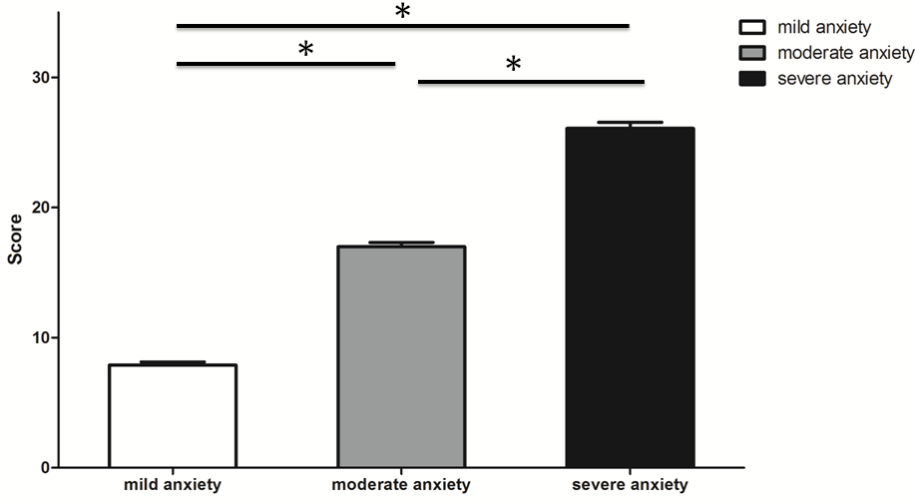
Test anxiety level according to Sarason TAS score Bars represent mean ± SD. * *p* < 0.05.

Students from both a specialized and a general school suffered from anxiety (*p* > 0.05): 35% had severe anxiety (TAS 26.09±3.87); 33% had moderate anxiety (16.98±2.64); and 32% had mild anxiety (7.89±1.92). These scores reflect significant differences (*p* < 0.05) (Figure 1). Thus, we found AS is a universal problem, independent of school type, which is consistent with other data showing that Asian students encounter high AS, even in adolescence [24].

### Telomere length

**Figure 2:**
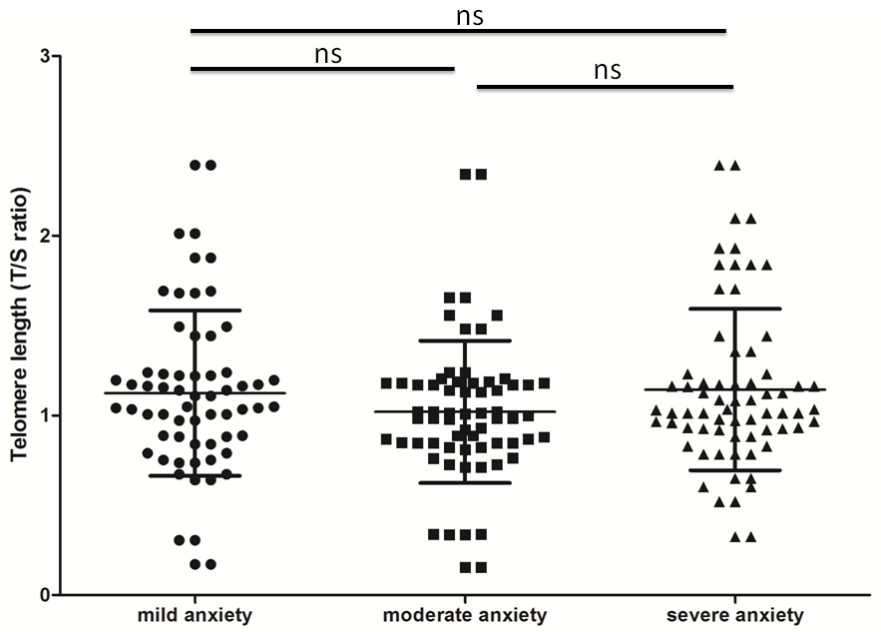
Telomere length (T/S ratio) of saliva samples according to test anxiety level Bars represent mean ± SD.

The average T/S ratio was 1.14 in students in both schools (range: 0.16 to 2.40). The three TAS subgroups had very similar TLs (Figure 2, *p* > 0.05); the T/S ratios were 1.14±0.46 (6.02 kb) in those with severe anxiety, 1.02±0.40 (5.74 kb) in those with moderate anxiety, and 1.12±0.45 (5.98 kb) in those with mild anxiety. There was no significant difference between males (1.06±0.40) and females (1.16±0.41) (*p* > 0.05) in this regard. After adjustment for gender, age, and BMI, the TLs of the three subgroups did not differ significantly (all *p* values > 0.05).

## DISCUSSION

AS is a severe problem in Asia, occurring earlier in life than in the West. AS affects even adolescents and is harmful to health [1, 25, 26]. We found, as expected, that students from both a specialized and a general school experienced AS. This underscores the inaccuracy of an idea commonly accepted in China: that students in general schools experience less AS because they are less concerned with academic accomplishment than are students in specialized schools. As it is true that students in specialized schools study hard and are more eager to enter prestigious universities than are students in general high schools, that even students in general schools experience AS shows that this problem is universal in China. Indeed, the early detection and treatment of severe AS is a critical public health issue.

Several methods are used to measure mean TL; these include terminal restriction fragment analysis by Southern blotting, quantitative PCR (qPCR), and fluorescence in-situ hybridization combined with flow cytometry (flow-FISH) [27, 28]. Southern blotting, which is the standard method, directly estimates both the average TL and the TL distribution. However, the assay has certain limitations, as it is time-consuming, requires a substantial amount of DNA, and overestimates the real lengths of telomeric sequences. qPCR and flow-FISH are simple and rapid and accurately measure TL. Gutierrez-Rodrigues et al. found that flow-FISH and qPCR were sensitive (both 100%) and specific (93% and 89%, respectively) when used to distinguish short telomeres from those of average length [29].

We measured the TL of cells in saliva rather then blood leukocytes because saliva collection is less invasive than blood collection; this is important when subjects are experiencing anxiety. Although the use of TL assays for blood leukocytes is a well-established technique, DNA from salivary epithelial cells can also be used to assess TL [30]. Mitchell et al. found a significant positive correlation (0.72; *p* = 0.002) between the TL of saliva and blood cells [31]. Emerging evidence shows that the extent of age-dependent telomere shortening is the same in cells of different tissues [32]. Moreover, the TL of epithelial cells predicts the development of age-related diseases, including oral and bladder cancer and Alzheimer's disease [33, 34]. Thus, our data are as reliable as any information that we could have obtained from leucocytes.

We found no significant TL shortening in response to AS in adolescent students facing their final examinations. Moreover, anxiety-caused TL shortening was not sex- or BMI-dependent, which is inconsistent with previous data [22, 23, 35]. This may be because our subjects were adolescents, and the previous studies involved adults. Moreover, our subjects were clinically normal, whereas most other studies enrolled psychiatric patients. Thus, the TLs measured in our study were not influenced by psychiatric treatment, which has been reported to cause telomere shortening [23]. Although our findings suggest that TL cannot serve as a biomarker of physical damage caused by psychiatric conditions, our data complement those of a major longitudinal study showing that a shorter TL in adults was associated with stress in early life [19, 36]. Very few studies have detected immediate telomere loss after psychological stress, although many have found that TL is a biomarker of chronological aging, and a shortened TL a risk factor for poor health outcomes after psychological stress early in life [37, 38]. The cited works focused on the impact of psychological stress during childhood or early life on adult TL [19, 36]. It is possible that AS may not negatively affect the TL until some time after the AS has dissipated. In mice, TL shortening caused by telomerase deprivation exhibits no phenotype prior to the third or fourth generation [39]. In agreement with the mouse data, telomerase-based in vitro cancer therapy afforded no anti-cancer effect until cells were subjected to several passages, indicating that telomere shortening has no effect for some time [40]. The cited works explain why stress in early life [41], or even intra-uterine stress [36], can shorten telomeres during adulthood but not in the concurrent negative environment. This also explains why the TLs of young adults with depression and anxiety did not significantly differ from those of controls [23]. It would be intriguing to examine how long the delayed effect of accelerated telomere loss persists even after an adverse environment is ameliorated. A large multi-center study is required to address this question. Moreover, a long-term longitudinal prospective study, rather than a study conducted at a single point in time, on the TLs of adults who experienced AS during their educational career may demonstrate that AS affects future health via telomere shortening.

## MATERIALS AND METHODS

### Participants

**Figure 3:**
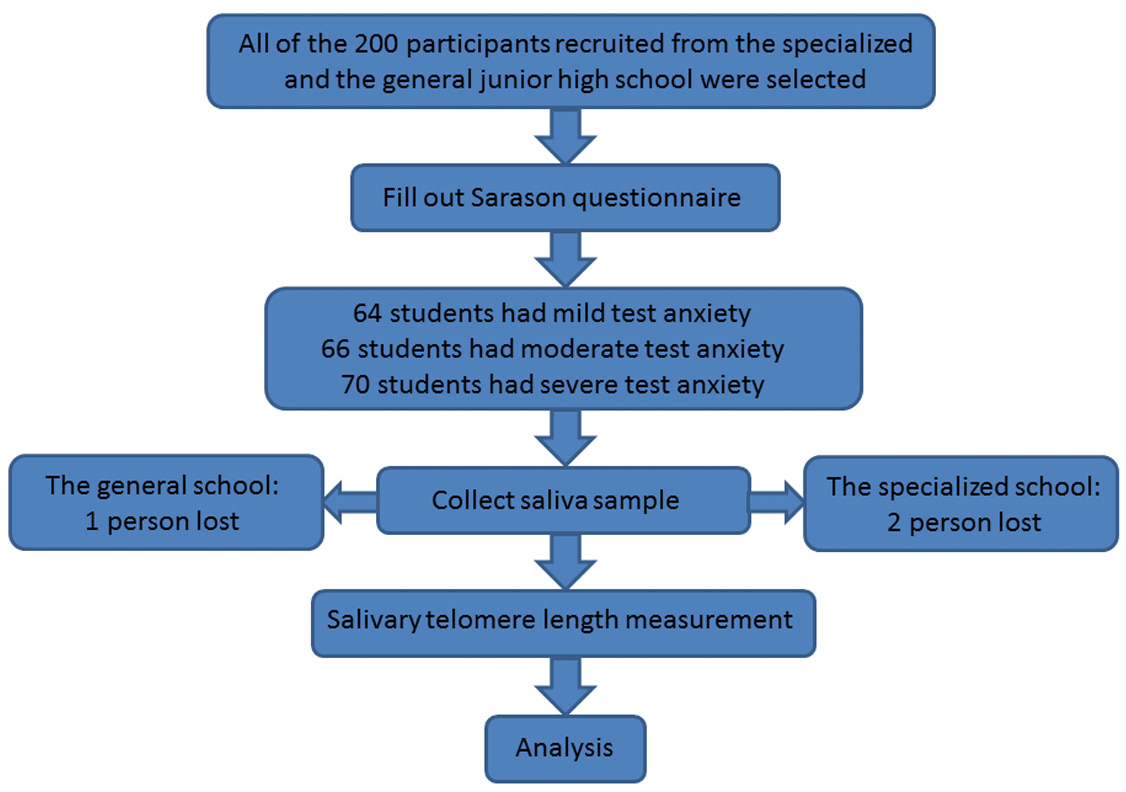
Flow diagram of the whole study procedure

Study participants were recruited in Shanghai, China via school newspapers. Before inclusion, all participants were screened via self-report and physical examination for acute or chronic health conditions, and those with such conditions were excluded. Interviewer-administered questionnaires were used to gather information on sociodemographic factors, psychosocial stress levels, and lifestyle factors. All subjects were non-smokers and were medication-free. Those meeting the DSM IV criteria for MDD were excluded [42]. In total, 200 adolescent students in grade 8 were ultimately recruited from either a specialized junior high school or a general school. Written informed consent was obtained from all participants and their parents. This study was approved by the Institutional Review Board of Shanghai Ruijin Hospital. Saliva samples were successfully collected from 98% of students in the specialized junior high school and from 99% of those in the general junior high school (Figure 3).

### AS measurement

The Sarason Test Anxiety Scale (TAS) was used to evaluate anxiety [43]. The TAS is reliable and yields valid data when used to evaluate Chinese populations [44]. The questionnaire features 37 no/yes questions scored as 0 or 1. To minimize self-selection bias and retrospective recall bias, we did not explain our hypotheses to the participants. All participants received the same (limited) information before recruitment. All experimenters were blind to subject anxiety status. The minimum and maximum test scores were 0 and 37, respectively; 0-12 indicates no or mild anxiety, 13-20 indicates moderate anxiety, and 21-37 indicates severe anxiety.

### Saliva collection

Saliva samples (2 mL) were collected into plastic tubes (Oragene Salivary kit, DNA Genotek Inc., Canada) in the morning (before breakfast) under the guidance of research personnel. The tubes were then sealed, releasing a stabilizing agent limiting DNA degradation and bacterial growth, and stored at -80°C prior to batch assay in the laboratory of Prof Yiming Lu at the Shanghai Ruijin Hospital.

### TL assays

Cells were collected from saliva samples using standard procedures, and DNA was extracted. TL was measured using a quantitative polymerase chain reaction (qPCR) method, and the ratio thereof to that of telomeres of standard reference DNA (the T/S ratio) was calculated as previously described [45]. The 36B4 gene served as a single-copy control gene. All assays were performed in triplicate. All DNA samples were coded, and laboratory staff were blind to subject identities. Conversion of the T/S ratios to base pairs was based on the mean telomeric restriction fragment length evident on Southern blotting. We plotted the slopes of the mean restriction fragment lengths against the T/S values using the following formula: Kilobase pairs (kb) = 3.274+2.413*(T/S) [46]. We confirmed that the assay sensitivity was adequate to detect TL variation before we analyzed the saliva samples. A result was considered acceptable if the control sample T/S ratio ranged from 0.95 to 1.05.

### Data analysis

We used the Shapiro-Wilk test to explore whether the salivary cell TL was normally distributed. Analysis of variance (ANOVA) was employed to compare differences in TAS scores and TLs by test anxiety levels. To explore whether gender, age, and/or BMI explained the associations between TAS scores and TL, we subjected these parameters to analysis of covariance (ANCOVA). The Friedman and Wilcoxon signed rank tests were used to compare nonparametric data. Normally distributed data are presented as means or percentages with standard deviations (SDs). All tests were two-sided, and the type I error rate was fixed at 0.05. All tests were performed with the aid of Statistical Analysis Software (SAS, version 9.3; SAS Institute, Cary, NC).

## References

[1] Liu Y, Lu Z (2012). Chinese high school students’ academic stress and depressive symptoms: Gender and school climate as moderators. Stress and Health.

[2] Dong M, Giles WH, Felitti VJ, Dube SR, Williams JE, Chapman DP, Anda RF (2004). Insights into causal pathways for ischemic heart disease adverse childhood experiences study. Circulation.

[3] Conley KM, Lehman BJ (2012). Test anxiety and cardiovascular responses to daily academic stressors. Stress and Health.

[4] Nanni V, Uher R, Danese A (2012). Childhood maltreatment predicts unfavorable course of illness and treatment outcome in depression: a meta-analysis. American Journal of Psychiatry.

[5] Ye J, Renault VM, Jamet K, Gilson E (2014). Transcriptional outcome of telomere signalling. Nature Reviews Genetics.

[6] Ye J, Lenain C, Bauwens S, Rizzo A, Saint-Léger A, Poulet A, Benarroch D, Magdinier F, Morere J, Amiard S (2010). TRF2 and Apollo cooperate with topoisomerase 2α to protect human telomeres from replicative damage. Cell.

[7] Azzalin CM, Reichenbach P, Khoriauli L, Giulotto E, Lingner J (2007). Telomeric repeat containing RNA and RNA surveillance factors at mammalian chromosome ends. Science.

[8] Robin JD, Ludlow AT, Batten K, Magdinier F, Stadler G, Wagner KR, Shay JW, Wright WE (2014). Telomere position effect: regulation of gene expression with progressive telomere shortening over long distances. Genes & development.

[9] Robin JD, Ludlow AT, Batten K, Gaillard MC, Stadler G, Magdinier F, Wright WE, Shay JW (2015). SORBS2 transcription is activated by telomere position effect-over long distance upon telomere shortening in muscle cells from patients with facioscapulohumeral dystrophy. Genome Research.

[10] Gilson E, Géli V (2007). How telomeres are replicated. Nat Rev Mol Cell Biol.

[11] von Zglinicki T (2002). Oxidative stress shortens telomeres. Trends in biochemical sciences.

[12] Choi J, Fauce SR, Effros RB (2008). Reduced telomerase activity in human T lymphocytes exposed to cortisol. Brain, behavior, and immunity.

[13] King KS, Kozlitina J, Rosenberg RN, Peshock RM, McColl RW, Garcia CK (2014). Effect of leukocyte telomere length on total and regional brain volumes in a large population-based cohort. JAMA neurology.

[14] Kao H, Cawthon R, Delisi L, Bertisch H, Ji F, Gordon D, Li P, Benedict M, Greenberg W, Porton B (2008). Rapid telomere erosion in schizophrenia. Molecular psychiatry.

[15] Verhoeven JE, Revesz D, Epel ES, Lin J, Wolkowitz OM, Penninx BW (2014). Major depressive disorder and accelerated cellular aging: results from a large psychiatric cohort study. Mol Psychiatry.

[16] Drury S, Theall K, Gleason M, Smyke A, De Vivo I, Wong J, Fox N, Zeanah C, Nelson C (2012). Telomere length and early severe social deprivation: linking early adversity and cellular aging. Molecular psychiatry.

[17] Mamdani F, Rollins B, Morgan L, Myers RM, Barchas JD, Schatzberg AF, Watson SJ, Akil H, Potkin SG, Bunney WE, Vawter MP, Sequeira PA (2015). Variable telomere length across post-mortem human brain regions and specific reduction in the hippocampus of major depressive disorder. Translational psychiatry.

[18] Gotlib IH, LeMoult J, Colich NL, Foland-Ross LC, Hallmayer J, Joormann J, Lin J, Wolkowitz OM (2015). Telomere length and cortisol reactivity in children of depressed mothers. Mol Psychiatry.

[19] Shalev I, Moffitt T, Sugden K, Williams B, Houts RM, Danese A, Mill J, Arseneault L, Caspi A (2013). Exposure to violence during childhood is associated with telomere erosion from 5 to 10 years of age: a longitudinal study. Molecular psychiatry.

[20] Schutte NS, Malouff JM (2014). The Relationship Between Perceived Stress and Telomere Length: A Meta-analysis. Stress and health.

[21] Hoen PW, Rosmalen JG, Schoevers RA, Huzen J, van der Harst P, de Jonge P (2013). Association between anxiety but not depressive disorders and leukocyte telomere length after 2 years of follow-up in a population-based sample. Psychological medicine.

[22] Shalev I, Moffitt TE, Braithwaite AW, Danese A, Fleming NI, Goldman-Mellor S, Harrington HL, Houts RM, Israel S, Poulton R, Robertson SP, Sugden K, Williams B (2014). Internalizing disorders and leukocyte telomere erosion: a prospective study of depression, generalized anxiety disorder and post-traumatic stress disorder. Mol Psychiatry.

[23] Needham BL, Mezuk B, Bareis N, Lin J, Blackburn EH, Epel ES (2015). Depression, anxiety and telomere length in young adults: evidence from the National Health and Nutrition Examination Survey. Mol Psychiatry.

[24] Tan JB, Yates S (2011). Academic expectations as sources of stress in Asian students. Social Psychology of Education.

[25] Singhal M, Manjula M, Vijay Sagar KJ (2014). Development of a school-based program for adolescents at-risk for depression in India: results from a pilot study. Asian journal of psychiatry.

[26] Park YJ, Shin NM, Han KS, Kang HC, Cheon SH, Shin H (2011). [Depression status of academic high school students in Seoul: mediating role of entrapment]. Journal of Korean Academy of Nursing.

[27] Baird DM (2005). New developments in telomere length analysis. Experimental Gerontology.

[28] Aubert G, Hills M, Lansdorp PM (2012). Telomere length measurement—Caveats and a critical assessment of the available technologies and tools. Mutation Research.

[29] Gutierrezrodrigues F, Santanalemos BA, Scheucher PS, Alvespaiva RM, Calado RT (2014). Direct comparison of flow-FISH and qPCR as diagnostic tests for telomere length measurement in humans. PloS one.

[30] Kroenke CH, Epel E, Adler N, Bush NR, Obradović J, Lin J, Blackburn E, Stamperdahl JL, Boyce WT (2011). Autonomic and adrenocortical reactivity and buccal cell telomere length in kindergarten children. Psychosomatic medicine.

[31] Mitchell C, Hobcraft J, McLanahan SS, Siegel SR, Berg A, Brooks-Gunn J, Garfinkel I, Notterman D (2014). Social disadvantage, genetic sensitivity, and children's telomere length. Proceedings of the National Academy of Sciences of the United States of America.

[32] Daniali L, Benetos A, Susser E, Kark JD, Labat C, Kimura M, Desai KK, Granick M, Aviv A (2013). Telomeres shorten at equivalent rates in somatic tissues of adults. Nature communications.

[33] Kemp RA, Reinders DM, Turic B (2007). Detection of lung cancer by automated sputum cytometry. Journal of Thoracic Oncology.

[34] Thomas P, O’Callaghan NJ, Fenech M (2008). Telomere length in white blood cells, buccal cells and brain tissue and its variation with ageing and Alzheimer's disease. Mechanisms of ageing and development.

[35] Müezzinler A, Zaineddin AK, Brenner H (2014). Body mass index and leukocyte telomere length in adults: a systematic review and meta-analysis. Obesity Reviews.

[36] Entringer S, Epel ES, Kumsta R, Lin J, Hellhammer DH, Blackburn EH, Wüst S, Wadhwa PD (2011). Stress exposure in intrauterine life is associated with shorter telomere length in young adulthood. Proceedings of the National Academy of Sciences.

[37] Blaze J, Asok A, Roth TL (2015). The long-term impact of adverse caregiving environments on epigenetic modifications and telomeres. Frontiers in behavioral neuroscience.

[38] Litzelman K, Witt WP, Gangnon RE, Nieto FJ, Engelman CD, Mailick MR, Skinner HG (2014). Association between informal caregiving and cellular aging in the survey of the health of wisconsin: the role of caregiving characteristics, stress, and strain. American journal of epidemiology.

[39] MaA Blasco, Lee H-W, Hande MP, Samper E, Lansdorp PM, DePinho RA, Greider CW (1997). Telomere shortening and tumor formation by mouse cells lacking telomerase RNA. Cell.

[40] Agrawal A, Dang S, Gabrani R (2012). Recent patents on anti-telomerase cancer therapy. Recent patents on anti-cancer drug discovery.

[41] Drury SS, Mabile E, Brett ZH, Esteves K, Jones E, Shirtcliff EA, Theall KP (2014). The association of telomere length with family violence and disruption. Pediatrics.

[42] Poling A, Methot LL, Lesage MG (1994). Diagnostic and Statistical Manual of Mental Disorders (DSM-IV). Jama.

[43] Sarason IG (1977). The Test Anxiety Scale: Concept and Research. DTIC Document.

[44] Shu S, Li TM, Fang FF, He HL, Zhou QH, Gu W, Zhou S (2011). [Relieving pre-exam anxiety syndrome with wrist-ankle acupuncture: a randomized controlled trial]. [Article in Chinese]. Zhong xi yi jie he xue bao.

[45] Boussouar A, Barette C, Nadon R, Saint-Leger A, Broucqsault N, Ottaviani A, Firozhoussen A, Lu Y, Lafanechere L, Gilson E, Magdinier F, Ye J (2013). Acacetin and chrysin, two polyphenolic compounds, alleviate telomeric position effect in human cells. Molecular therapy Nucleic acids.

[46] Entringer S, Epel ES, Kumsta R, Lin J, Hellhammer DH, Blackburn EH, Wust S, Wadhwa PD (2011). Stress exposure in intrauterine life is associated with shorter telomere length in young adulthood. Proceedings of the National Academy of Sciences of the United States of America.

